# The Retort

**Published:** 1891-09

**Authors:** Geo. P. Morris


### THE RETORT.

Old Nick, who taught the village school,
Wedded a maid of homespun habit;
He was stubborn as a mule,
She was playful as a rabbit.
* * * *
One day the tutor went abroad,
And simple Jennie sadly missed him;
When he returned, behind her lord
She slyly stole, and fondly kissed him!

The husband's anger rose!—and red
And white his face alternate grew!
"Less freedom, ma'am;" Jane sighed and said,
"*Oh, dear! I didn't know 'twas you!*"

—By GEO. P. MORRIS.

